# Primary Sternal Osteomyelitis or Sternal Pseudotumor of Childhood: A Case Series and Review of the Literature

**DOI:** 10.1097/INF.0000000000005213

**Published:** 2026-03-10

**Authors:** Niklaus Schoepke, Tobias Krause, Kai Ziebarth, Milan Milosevic

**Affiliations:** From the * Pediatric Surgery Department, University Children’s Clinic Bern, Bern, Switzerland; † Pediatric Orthopedic Department, University Children’s Clinic Bern, Bern, Switzerland.

**Keywords:** osteomyelitis, pseudotumor, sternum, children

## Abstract

**Background:**

Primary sternal osteomyelitis (PSO) in children is a rare condition with less than 100 cases described in the literature. Sternal pseudotumors and Self Limiting Sternal Tumor of Childhood (SELSTOC) as a different entity seem to have similar clinical findings but a completely different approach to treatment.

**Methods:**

We analyze a case series of 5 cases of PSO and review the literature to compare PSO and SELSTOC.

**Results:**

Main symptoms of PSO and SELSTOC involve presternal swelling (90% vs. 100%), pain (70% vs. 58%), erythema (47% vs. 27%) and fever (60% vs. 21%). The most reliable laboratory marker is erythrocyte sedimentation rate (96% vs. 100% positive), followed by C-reactive protein (89% vs. 65% positive) and white blood cell count (64% v.s 45% elevated). A pathogen could be isolated in 88% of PSO cases, whereas only in 6% of SELSTOC cases. While intervention or surgery rates were similar (58% vs. 38%), treatment with antibiotics differed significantly (100% vs. 33%).

**Conclusions:**

Considering the similarities in clinical and radiologic features, SELSTOC might be a phenotype of PSO rather than a separate entity. Improved pathogen detection may help clarify its relationship to PSO and guide appropriate management strategies, particularly to avoid unnecessary treatment in benign cases of PSO.

Primary sternal osteomyelitis (PSO) is a hematogenous infection of the sternum not caused by a penetrating trauma or sequela of surgery of adjacent organs. It is a relatively rare condition in children, with only 100 cases described in the literature up to this date.^1–3^ The peak incidence is in early childhood at a median age of 1 year, and a few cases are reported in adolescents.^1^ Its presentation is often nonspecific, with only a few cases showing all clinical cardinal symptoms of inflammation and elevated laboratory inflammation markers. With the most common symptom of presternal swelling, PSO shares the same clinical manifestation with seemingly another entity described in the literature. Various authors described multiple cases of infants with presternal swelling and additional clinical symptoms of inflammation in infants with an age peak of 1–2 years and referred to them as “Self Limiting Sternal Tumor of Childhood (SELSTOC)” or “Sternal Pseudotumor (of Childhood).”^4–6^ In this article, we present 5 cases of pediatric PSO and compare their findings with those previously described in the literature as PSO and as Sternal Pseudotumor/SELSTOC.

## METHODS

We conducted a retrospective analysis of all the patients presenting with findings consistent with PSO in our tertiary pediatric hospital from 2013 to 2024. The patients were identified through a search of our clinic’s electronic database and imaging data from the radiology information system. Data on patient demographics, clinical findings, laboratory inflammation markers, microbiology and radiology were then collected and analyzed retrospectively. Molecular diagnostics were performed using a broad-range bacterial polymerase chain reaction (PCR) targeting the 16S rRNA gene on clinical specimens, followed by sequencing for pathogen identification. The results are depicted in Table 1.

**TABLE 1.** Patient Characteristics

Patients	1	2	3	4	5
Age (months)	12	13	22	12	19
Duration of symptoms (d)	5	3	17	5	6
Swelling	Yes	Yes	Yes	Yes	Yes
Erythema	Yes	No	Yes	Yes	no
Pain	Yes	Yes	Yes	Yes	No
Fever	No	Yes	No	Yes	No
CRP (mg/L)	18	3	3	26	53
WBC (G/L)	6.6	7.5	8.2	10.4	16.2
ESR (mm/h)	110	38	28	60	63
Blood culture	Negative	Negative	Negative	Negative	Negative
Intraoperative specimen	Positive	Positive	Positive	Negative	Positive
Isolated microorganism	*K. kingae*	*K. kingae*	*K. kingae*	None	*K. kingae*
Ultrasound	Yes	Yes	Yes	Yes	Yes
Radiograph	Yes	No	No	Yes	Yes
MRI	Yes	No	Yes	Yes	Yes
Surgery	Incision, Curretage, Gentamicin sponge	Incision, Curretage, Gentamicin sponge, twice	Incision, Curretage, Gentamicin sponge	Incision, Curretage, Gentamicin sponge	Incision, Curretage, Gentamicin sponge
Antibiotic treatment	Cefuroxime	Cefuroxime	Cefuroxime	Amoxicillin/clavulanic acid	Cefuroxime
Antibiotic treatment duration	5d i.v., 22d p.o.	3d i.v., 28d p.o	5d i.v., 21d p.o	5d i.v., 21d p.o	5d i.v, 20d p.o

For the review, a literature search was conducted in PubMed and Google Scholar using the terms “sternal osteomyelitis,” “primary osteomyelitis,” “osteomyelitis,” “sternum” and “sternal” in combination with pediatric age terms “child,” “pediatric,” “infant,” “adolescent” for the PSO cases. Because Schweitzer et al.^1^ did such a thorough review, we collected all the cases since the publication of their paper and added the findings to their results. For the SELSTOC cases, we used the terms “SELSTOC,” “Self limiting Sternal Tumor,” “Sternal Pseudotumor,” “Sternal Swelling” combined with the pediatric age terms. Like our cohort, we collected data on patient demographics, clinical findings, laboratory inflammation markers, microbiology and radiology. We used age-corrected values to evaluate the laboratory markers. Because in some studies, laboratory markers were only labeled as positive and negative, we did not specify any mean or median values and ranges. We then compared the findings of the data collected from PSO cases and SELSTOC cases (Table 2).

**TABLE 2.** Comparison of Findings in Patients of Primary Sternal Osteomyelitis (PSO) to Self-Limiting Sternal Tumors of Childhood (SELSTOC)

	PSO	SELSTOC
Age (years)	1.16	1.00
Male	65%	57%
Clinical symptoms		
Swelling	90%	100%
Tenderness	70%	58%
Erythema	47%	27%
Fever	60%	21%
Laboratory findings		
WBC elevated	64%	45%
CRP elevated	89%	65%
ESR elevated	96%	100%
Imaging		
Plain radiograph	66%	71%
Abnormal	55%	63%
Osteolysis	37%	21%
Ultrasound	34%	91%
MRI	27%	21%
CT	13%	19%
Pathogen isolated	88%	6%
Intervention	58%	38%
Antibiotic treatment	100%	33%
Resolve	100%	100%

*P* values were not calculated due to inconsistent and incomplete reporting of findings in the literature

## RESULTS

### Case Series

#### Patients

A total of 5 patients with the diagnosis of PSO were treated from 2013 to 2024, making up 2.5% of all children with primary osteomyelitis seen at our clinic. The median age was 13 months (12–19, mean 15.6 months) with a female to male ratio of 3:2. All children were previously healthy with no underlying chronic condition.

#### Clinical and Laboratory Findings

**FIGURE 1.**
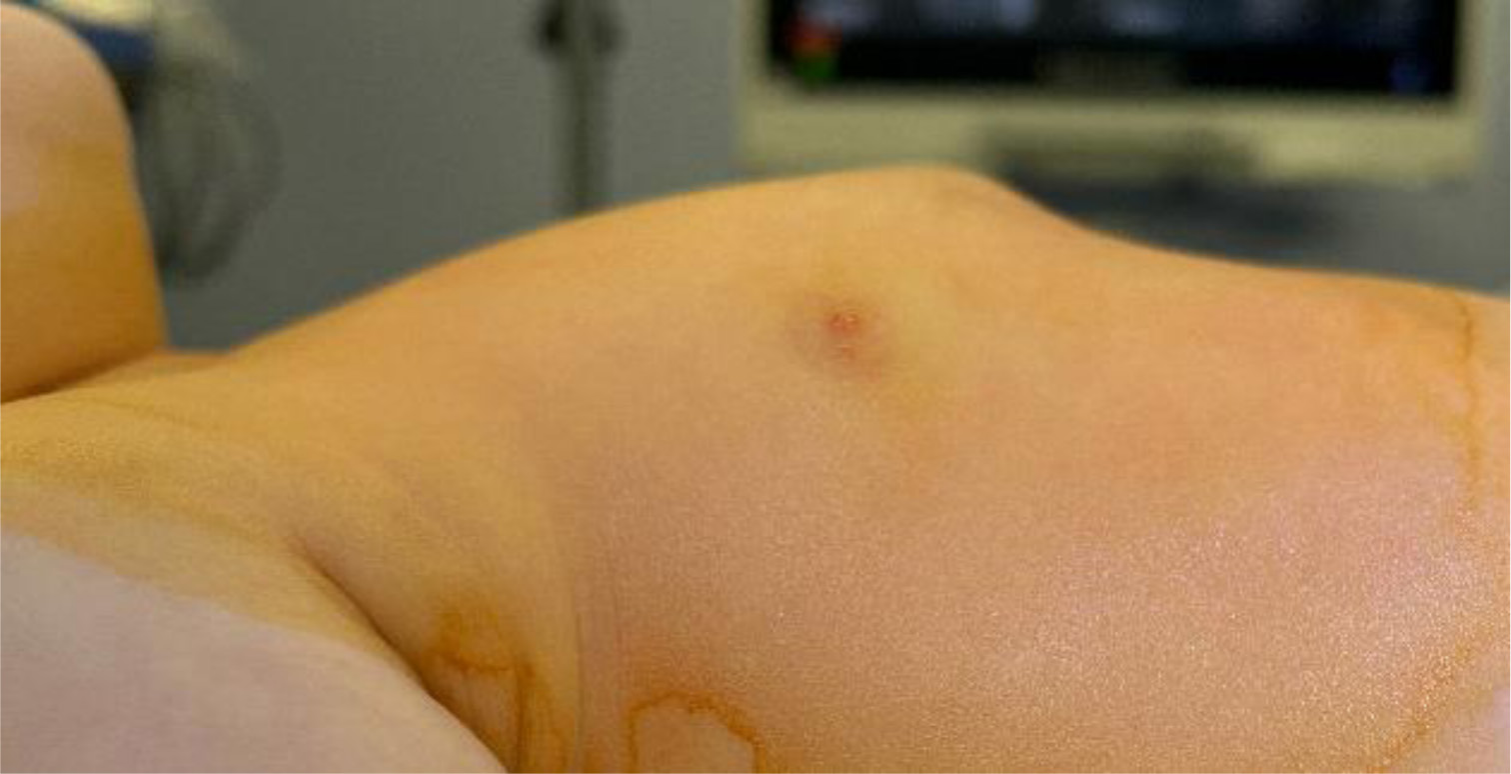
Clinical appearance before surgery.

The main symptoms were presternal swelling (100%), presternal tenderness (80%) and presternal erythema (60%); fever was only present in 2 of 5 patients (40%). Median time from onset of symptoms to diagnosis was 5 days (range 3–17 days, mean 7.2 days) (Fig. 1).

Laboratory investigations showed an elevated CRP in 2 patients (median 18 mg/l, range 3-53 mg/l). Leukocytosis was present in only one patient (median 8.3 G/L, range 6.6–16.2 G/L). All patients had an increased erythrocyte sedimentation rate (ESR) (median 60 mm/hour, range 28–110 mm/hour). Thrombocytosis was found only in 1 patient (median 395 G/L, range 309–665G/L).

#### Imaging

Conventional radiography was performed in 3 patients; in 2 of these, abnormal findings were observed, including presternal swelling and osteolysis of the sternal body.

**FIGURE 2.**
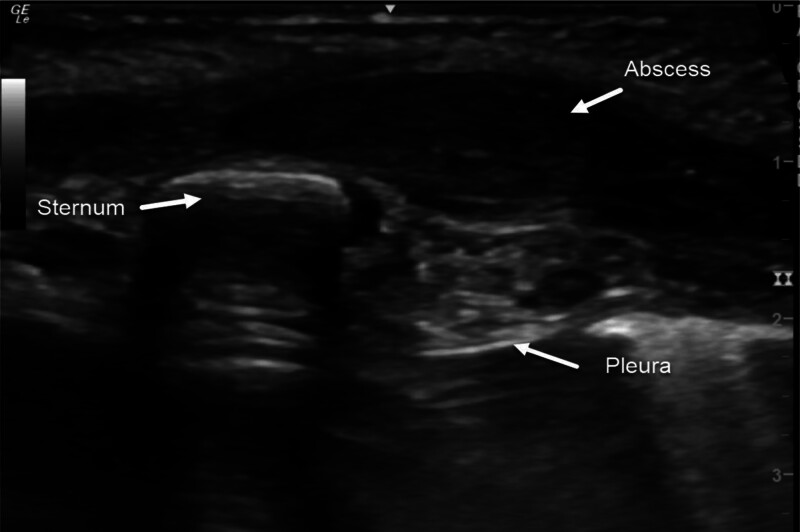
Axial ultrasound showing hypoechogenic mass presternal, suggesting abscess formation with tissue alteration reaching the retrosternal space.

All 5 patients underwent sonography, which revealed pathological findings in every case. The imaging showed a heterogeneous, hypoechoic mass, which in some cases had a dumbbell shape extending from the presternal region through the ossification centers of the sternum to the retrosternal space. The largest measured diameter ranged from 13 to 25 mm (Fig. 2).

**FIGURE 3.**
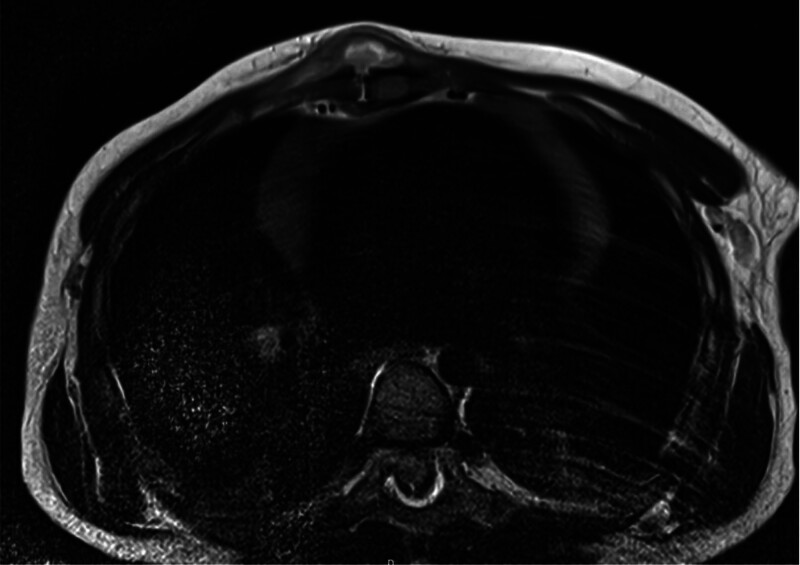
Axial MRI showing presternal abscess formation reaching through to the retrosternal space.

**FIGURE 4.**
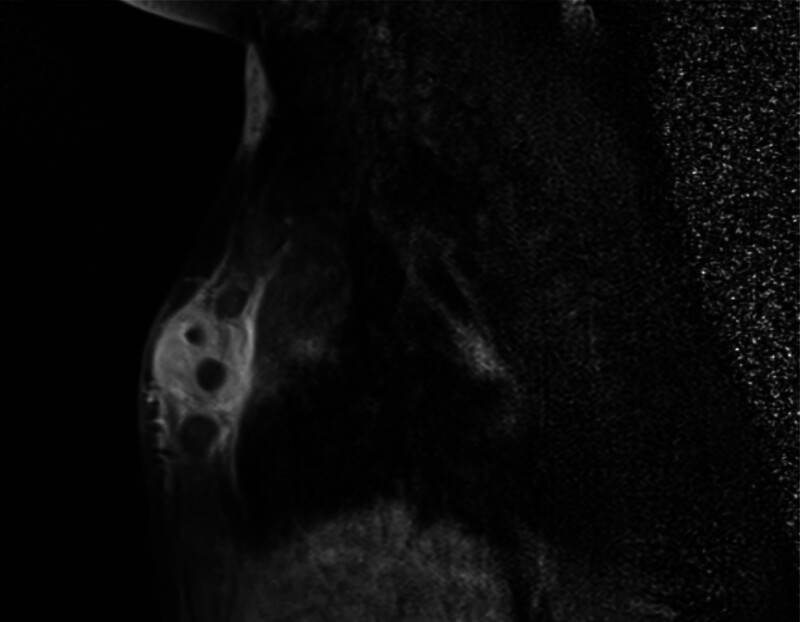
Sagittal MRI showin presternal mass reaching the retrosternal space, widening of gaps between sternal bony bodies.

Four of 5 patients had an additional magnetic resonance imaging (MRI). All cases showed bony involvement of the sternum, including bone marrow edema and some degree of cortical destruction. Each patient had a lesion with central diffusion restriction and peripheral contrast enhancement, interpreted as an abscess. This was located either in the bone, subperiostal or in the presternal tissue. Pre- and retrosternal soft tissue swelling, often with perifocal edema and strong post-contrast enhancement, was shown in all cases. There was no significant lymphadenopathy found, with only mild pleural effusion or atelectasis due to sedation in some cases (Figs. 3 and 4).

#### Treatment

All 5 patients underwent surgery after diagnosis of PSO. There was no liquid pus found in any of the patients. Surgeons described the intraoperative findings as “disintegrated soft tissue,” “tissue of gelatinous consistency,” “yellowish caseous necrosis” and “encapsulated, firm tissue” with various degrees of bony destruction. After debridement and rinsing the situs, a gentamicin-coated collagen sponge was placed in all patients.

Patient 2 underwent a second surgery after 16 days because of persistent clinical swelling and progression of subcutaneous collection on ultrasound. The surgeon again described no liquid pus but “cell detritus.” The surgical intervention consisted of debridement, rinsing and placement of a gentamicin-coated collagen sponge.

All patients received postoperative antibiotic treatment for a total of 4 weeks (median 26 days, range 25–31 days). Four patients were treated with cefuroxime; 1 patient who was transferred to our hospital was continued with amoxicillin/clavulanic acid, which was already administered for 2 days before surgery.

After 4 weeks of treatment, complete resolution was seen in all patients.

#### Histologic and Microbiologic Findings

Blood culture was sterile in all patients. *Kingellae kingae* was detected in 4 patients in the tissue via PCR. No microorganism could be detected in the tissue from the patient who was already treated with antibiotics 2 days before surgery.

Histology showed chronic, fibrotic and acute inflammation in all patients; 4 patients showed purulent inflammation; and 1 showed tissue necrosis. No signs of malignancy were found in any of the cases.

### Analysis of Literature

#### Primary Sternal Osteomyelitis

A total of 74 cases were identified by Schweitzer et al.,^1^ 13 cases of those stem from the preantibiotic and 61 cases from the antibiotic era. Since their review, another 25 cases were published including our case series.^2,7–16^ Of those 25 patients, 9 patients could not be further analyzed since they were part of a series of osteomyelitis in flatbones^15^ or osteoarticular infections of the chestwall,^16^ and the authors did not elaborate the results separately. This results in a total of 77 cases in the antibiotic era.

Median age is 1.16 years with a range from 10 days to 18 years. There is an age peak at the age around 1 year and a much smaller one in adolescence. Males are twice as often affected as females (47:25). Only 7 patients had previous conditions, 1 patient with systemic lupus and 6 patients with sickle cell disease.

Clinical symptoms involved presternal swelling (90%), tenderness (70%) and erythema (47%). Fever was reported in 60% of the children.

White blood cell count was performed in 88% of the patients and was elevated in 64% of those cases. C-reactive protein (CRP) was reported in 58% of the patients and was positive in 89%. ESR was specified in 58% of the patients as well, and elevated in 96%.

Imaging was performed using plain radiographs in 66% of the patients. Of all the 51 radiographs, 28 radiographs (55%) were abnormal, with 19 radiographs (37%) showing signs of osteolysis of the sternum. Ultrasound was done in 34% of the patients; findings included thickened periosteum and destruction of the cortex, subcutaneous and mediastinal hypoechogenic masses, sometimes connected, showing the image of a dumbbell, abscess formation in the sternum and adjacent soft tissue reactions. Further imaging studies included MRI in 27% of the patients and a CT scan in 13%.

A pathogen could be identified in 88% of the cases. Of those 67 cases, the pathogen was isolated in blood culture alone in 22% of the patients, in tissue culture alone in 63%, and in both tissue and blood culture in 18% of the patients. Seven percent of the cases could only be detected by PCR in blood or tissue samples, all of them being infections caused by *K. kingae. Staphylococcus aureus* was the most frequent causative microorganism for PSO, found in 52% of cases, with a total of 7% being methicillin-resistant *S. aureus* (MRSA). *Kingella kingae* was the second most detected microorganism in 18% of the patients, exclusively in patients 36 months or younger. *S. Salmonella* was found in 10% patients, 5 of whom suffered from sickle cell disease and 2 came from rural China, being exposed to contaminated food and water as the author suggests.^9^ Coagulase-negative *Staphylococcus* was detected in 5% and *Streptococcus pneumoniae* in 3% of the patients. There was 1 case each of infections with Group B *Streptococcus, Brevibacterium*, *Brucella melitensis* and *Treponemapallidum.*

Interventions ranging from biopsy to partial resection of the sternum were reported in 58% of the cases, with the most frequent intervention being incision and debridement. All patients received antibiotic treatment with a duration ranging from 2 weeks to 1 year (median 5 weeks). The antibiotics used differed widely, from staphylococci specific to broad-spectrum antibiotics.

In all patients, the osteomyelitis was resolved after treatment. One patient died, according to the authors, probably due to a complication from a concomitant disease rather than the osteomyelitis itself.^1^ Since the review from Schweitzer et al., there were no more long-term complications reported, leaving the 2 reports with osteolysis and a divided sternum as the sole long-term complications.

#### Sternal Pseudotumor/SELSTOC

A total of 14 publications were identified describing sternal pseudotumor in children, as well as the entity termed SELSTOC by te Winkel et al., encompassing 42 patients in total.^4–6,17–27^

Median age was 1.0 years with a range from 1 month to 2.7 years. Males were more often affected than females (24:18). All patients were previously healthy.

Presternal swelling was present in all patients, tenderness in 58% (6 patients without information), erythema in 27% (4 patients without information) and fever in 21% patients.

Laboratory findings included white blood cell count performed in 69% of the patients and positive in 45% of those patients. CRP was specified in 62% of the cases and elevated in 65%. ESR was evaluated in 38% of the patients and positive in all of them.

Plain radiographs were performed in 30 (71%) of the patients. The results of 11 radiographs were not further disclosed. Of the remaining 19 radiographs, 12 radiographs (63%) showed abnormal findings, 4 of those radiographs (21%) showing signs of osteolysis of the sternum. Ultrasound was conducted in 91% of the patients. Ultrasound findings were described as typically a dumbbell-shaped heterogenic hypoechoic mass spanning from presternally to retrosternally with various degrees of vascularity, sometimes accompanied by bony destruction and abscess formation. Further imaging was done in 21% of the patients using MRI and 19% of the patients using a CT scan.

Microbiologic examinations were reported in 17 (40%) of the cases. In only 1 patient, both blood and tissue samples were positive for *S. aureus* (6%); all other examinations remained negative.

Interventions were performed in 16 of the cases (38%). The intervention consisted of aspiration/biopsy in 7 patients (17%) and incision and debridement/drainage in 9 patients (21%). Fourteen (33%) of the patients received antibiotic treatment; only in 3 cases, the duration was described (2 patients 1 week, 1 patient only 2 days).

Histologic findings reported in 9 cases found signs of active and chronic inflammation in all the samples.

The process resolved in all cases, and there were no long-term sequalae described.

## DISCUSSION

In this study, we are adding 5 pediatric cases of PSO to the limited number reported in the literature. When compared to all the cases reported in the literature, our patients showed similar clinical features, with various combinations of the cardinal symptoms of inflammation in the sternal area. Fever is not a mandatory symptom for the differential diagnosis of PSO, being reported only in 60% of all cases.

The most sensitive laboratory inflammation marker in PSO seems to be ESR, with abnormal values in all our cases and 96% in the literature. While plain radiographs were the most used imaging studies, only 37% were suspicious or diagnostic for osteomyelitis. With ultrasound now almost everywhere available and without ionizing radiation, it is questionable if plain radiographs add value to the differential diagnosis. In all our cases, ultrasound showed more detailed involvement of bone and surrounding tissue, as it is also described in the literature. With PSO still being a rare disease, MRI was performed in 4 of 5 patients in our series, confirming the diagnosis and showing the extension of the process. However, all our patients needed sedation for further imaging using MRI.

*Staphylococcus aureus* was still the most frequent microorganism causing PSO. The most sensitive way of pathogen detection was tissue sampling, with blood culture only being positive in 22% of the patients. *Kingella kingae* was the second leading microorganism in all cases of the literature and found in 4 of our cases; all patients were under the age of 3 years. In our series, *K. kingae* was identified only via PCR, highlighting the potential for underdiagnosis when relying solely on culture. Considering that pathogens were reported in 88% of published cases, the absence of PCR in many studies may mean the true prevalence of *K. kingae* has been underestimated. Therefore, the use of PCR or adequate systems to detect *K. kingae* should be included in the diagnostic process of sternal osteomyelitis, especially in infants. This was also shown in a recent multinational study by Olijve et al.^28^ An additional noninvasive tool to help diagnose *K. kingae*-related osteoarticular infections in children under 48 months of age can be an oropharyngeal swab PCR assay for *K. kingae*, with a reported sensitivity and specificity of 100% and 90.5%, respectively.^29^

In our case series, as well as some cases reported in the literature, osteolytic lesions are seen on imaging with a relatively short period of symptoms before presentation. It seems reasonable to assume that, therefore, the infection must have been going on for a longer period without any obvious signs of inflammation. In view of this, as well as the often very mild symptoms, PSO seems to present often as subacute osteomyelitis as described by King and Mayo.^30^ This also aligns with the study of Spyropoulou et al., which found that ESR is the most sensitive laboratory inflammation marker in subacute osteomyelitis and blood cultures, as well as cultures from specimens often remain negative when not specifically tested for *K. kingae*.^31^ As a flat bone lacking physis-associated slow blood flow facilitating bacterial seeding as well as being supplied by a rich anastomotic arterial network, the sternum may benefit from enhanced immune surveillance and early containment of hematogenous infection. These features may limit both the establishment and progression of infection, potentially accounting for the predominance of subacute forms.

All our patients underwent surgery, with 1 patient having a second intervention due to persisting changes in ultrasound after 2 weeks. After 4 weeks of antibiotic treatment, complete resolution was seen in all our patients. In the literature, 42% of patients healed without any intervention. These findings indicate that surgical intervention may not be necessary in all cases and could be considered primarily for patients with severe clinical presentation or lack of response to antibiotic treatment.

In 2009, te Winkel et al. first defined the term self-limiting sternal tumors of childhood (SELSTOC) by investigating their own cohort of 14 cases of a presternal tumor presenting to a pediatric oncology center and comparing them to similar cohorts of Howard et al. and Roukema et al. Due to a favorable outcome without special treatment, the authors proposed a wait-and-see approach for children presenting with this entity. Since then, various authors have described similar cases under the name SELSTOC or sternal pseudotumors, advocating for sonography as the main diagnostic tool and acknowledging the conservative treatment.

When comparing these patients to the PSO cases, we found striking similarities in patient presentation and diagnostics but significant differences in treatment (Table 1).

The median age as well as the gender relation is almost the same; the only difference is seen in age distribution, with none of the SELSTOC patients being older than 3 years. Clinical symptoms also showed various combinations of local inflammation signs, but with fewer cases reported having fever in the SELSTOC group.

As well as in PSO patients, ESR was the most sensitive laboratory inflammation marker in SELSTOC patients. In SELSTOC patients, ultrasound was the most used imaging method, showing the same pathological findings as were described in the PSO group. Radiographs were similarly used and showed similar results, including the rate of osteolysis. The use of further imaging studies was also comparable.

Pathogen isolation was significantly lower in SELSTOC patients with a detection rate of 3% compared to 88% in PSO patients. One plausible reason might be a limited use of adequate modalities for the detection of *K. kingae*, potentially leading to underestimation of its prevalence in SELSTOC patients, especially considering the age distribution of under 3 years.

The rate of surgical interventions was higher in PSO patients with 58% compared to 38% in the SELSTOC group, with more extensive and invasive surgeries reported in PSO patients. Histological findings showed the same changes in SELSTOC patients as reported in our cohort.

The use of antibiotics was significantly lower in the SELSTOC group, with 33% of the patients receiving antibiotic treatment compared to 100% of the PSO patients. Unfortunately, the duration of antibiotic treatment was only declared in 3 patients in the SELSTOC group. But the stated duration in those two cases and the narrative details imply that these courses were likely shorter compared to those of the PSO patients.

## CONCLUSION

The similarities between SELSTOC and PSO raise the question of whether SELSTOC is indeed a distinct condition or instead reflects a milder phenotype of PSO in younger children, caused by *K. kingae* or other low-pathogenic pathogens, and therefore not being detected when not specifically searched for. However, if this were the case, the observation that approximately 67% of SELSTOC patients recovered without antibiotic therapy, could indicate that current treatment practices for PSO in this specific age group may be more intensive than necessary. Further research and reporting of this condition are necessary to find a proper treatment algorithm and prevent unnecessary diagnostics, interventions and prolonged antibiotic treatment.
